# Fluorescence-guided surgery of a highly-metastatic variant of human triple-negative breast cancer targeted with a cancer-specific GFP adenovirus prevents recurrence

**DOI:** 10.18632/oncotarget.12314

**Published:** 2016-09-28

**Authors:** Shuya Yano, Kiyoto Takehara, Shinji Miwa, Hiroyuki Kishimoto, Hiroshi Tazawa, Yasuo Urata, Shunsuke Kagawa, Michael Bouvet, Toshiyoshi Fujiwara, Robert M. Hoffman

**Affiliations:** ^1^ AntiCancer, Inc., San Diego, CA, USA; ^2^ Department of Surgery, University of California San Diego, CA, USA; ^3^ Department of Gastroenterological Surgery, Okayama University Graduate School of Medicine, Dentistry and Pharmaceutical Sciences, Okayama, Japan; ^4^ Center for Innovative Clinical Medicine, Okayama University Hospital, Okayama, Japan; ^5^ Oncolys BioPharm Inc., Tokyo, Japan

**Keywords:** fluorescence-guided surgery (FGS), telomerase dependent, adenovirus, GFP/RFP, survival

## Abstract

We have previously developed a genetically-engineered GFP-expressing telomerase-dependent adenovirus, OBP-401, which can selectively illuminate cancer cells. In the present report, we demonstrate that targeting a triple-negative high-invasive human breast cancer, orthotopically-growing in nude mice, with OBP-401 enables curative fluorescence-guided surgery (FGS). OBP-401 enabled complete resection and prevented local recurrence and greatly inhibited lymph-node metastasis due to the ability of the virus to selectively label and subsequently kill cancer cells. In contrast, residual breast cancer cells become more aggressive after bright (white)-light surgery (BLS). OBP-401-based FGS also improved the overall survival compared with conventional BLS. Thus, metastasis from a highly-aggressive triple-negative breast cancer can be prevented by FGS in a clinically-relevant mouse model.

## INTRODUCTION

Triple-negative breast cancers (TNBC) are defined by a lack of expression of estrogen, progesterone, and ERBB2 receptors, and are the most aggressive and metastatic breast cancer. The absence of effective treatment for this recalcitrant disease lead to a higher rate of local and systemic relapse after primary tumor resection.

Fluorescence-guided surgery (FGS) is an area of intense research [1]. Green fluorescent protein (GFP) has been previously successfully used for targeting tumors *in situ* for FGS using a telomerase-dependent adenovirus (OBP-401) that expresses the *gfp* gene only in cancer cells [1–7]. Since recurrent cancer cells stably express GFP, detection of cancer recurrence and metastasis is also possible with OBP-401 GFP labeling [7], in contrast to fluorescent-antibody or other non-genetic labeling [1].

We have previously demonstrated OBP-401-based fluorescence-guided surgery is highly effective in various types of cancers including soft tissue sarcoma [4], glioblastoma [5], pancreatic cancer [6], lung cancer [7], colon cancer-liver metastasis [8], and melanoma [9]. In contrast, conventional bright-light surgery (BLS) could not fully resect these tumors [4–9].

In the present study, we describe that OBP-401 targets a high-metastatic variant of triple-negative human breast cancer, an aggressive recalcitrant cancer, in nude mice and enables complete resection, prevents recurrence and improves disease-free survival and over-all survival. The clinical implications of these results are discussed.

## RESULTS AND DISCUSSION

### GFP-expressing adenovirus OBP-401 labels low- and high-invasive breast cancer cells *in vitro*

**Figure 1:**
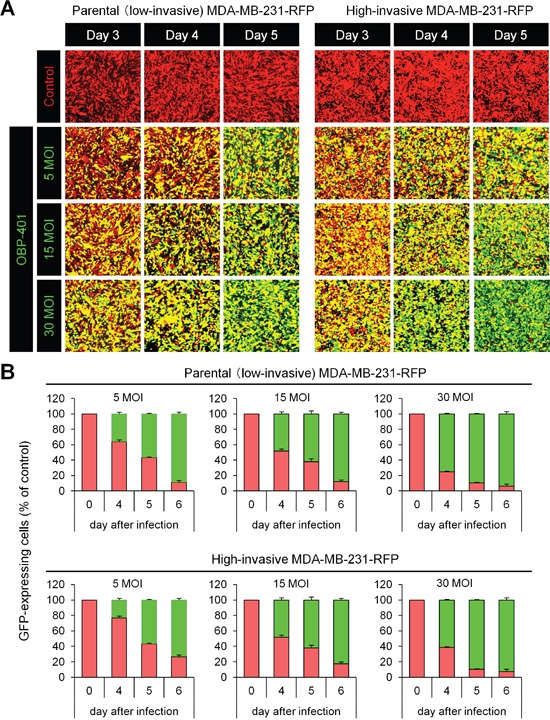
OBP-401 labels low- and high-invasive MDA-MB-231-RFP breast cancer cells *in vitro* Low- and high-invasive variants of MDA-MB-231-RFP cells were seeded in 6-well plates (1 × 10^5^ cells a well). OBP-401 was added at the indicated multiplicity of infection (MOI) 24 hours after seeding. Images were acquired with a confocal laser scanning microscope FV1000 (Olympus, Tokyo, Japan). **A.** Representative images of low- and high-invasive MDA-MB-231-RFP breast cancer cells 3, 4, and 5 days after infection of OBP-401 at the indicated MOI **B.** Histogram shows the frequency of GFP-expressing parental and highly metastatic MDA-MB-231-RFP breast cancer cells at indicated days after infection of OBP-401. The number of GFP-expressing cells was counted. Data are shown as average ± SD. N = 5.

Firstly, we confirmed whether OBP-401 could label low- (parental) and high-invasive MDA-MB-231-RFP breast cancer cells *in vitro*. Time course imaging showed that OBP-401 labeled low- and high-invasive MDA- MB-231-RFP breast cancer cells with GFP (Figure 1A). OBP-401 labeled low- and high-invasive MDA-MB-231- RFP breast cancer cells in a dose-dependent manner (Figure 1B).

### Bright-light surgery results in residual cancer cells

**Figure 2:**
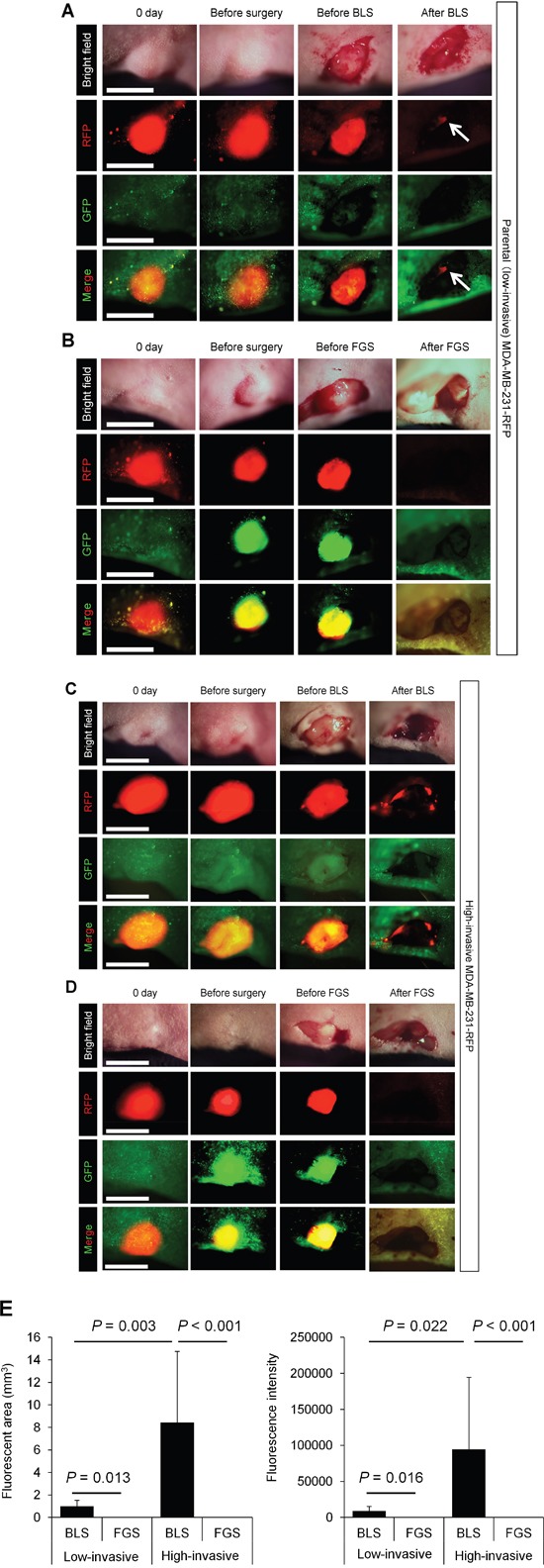
Comparison of OBP-401-based fluorescence-guided surgery with bright-light surgery for orthotopic low- and high-invasive MDA-MB-231-RFP OBP-401 was injected intaratumorally at 1 × 10^8^ PFU when tumors reached approximately 10 mm^3^ (diameter; 3 mm). **A.** Representative whole-tumor images of low-invasive MDA-MB-231-RFP cells before and after bright light surgery (BLS). White arrows point to the residual tumor. **B.** Representative whole-tumor images of low-invasive MDA-MB-231-RFP before injection of OBP-401, before and after OBP-401-based fluorescence-guided surgery (OBP-401 FGS). **C.** Representative whole-tumor images of high-invasive MDA-MB-231-RFP before and after BLS. **D.** Representative whole-tumor images of high-invasive MDA-MB-231-RFP before injection of OBP-401 and before and after OBP-401-based fluorescence-guided surgery (OBP-401 FGS). **E.** Histogram shows the comparison of fluorescent area (left) and fluorescence intensity (right) of residual tumor in the surgical bed after BLS or OBP-401 FGS of low- and high-invasive MDA-MB-231-RFP. Fluorescence intensity and fluorescent area were calculated with ImageJ software. Data are shown as average ± SD. N = 10.

We performed bright (white) light surgery (BLS) on orthotopic low- and high-invasive MDA-MB-231-RFP in nude mice (Figures 2A, 2C). High-invasive MDA-MB-231-RFP cells invaded the normal mammary gland, making the tumor margin invisible, thereby resulting in many cancer cells in the surgical field after BLS (Figure 2C).

### OBP-401-based fluorescence-guided surgery (OBP-401 FGS) enables resection of residual cancer cells

**Figure 3:**
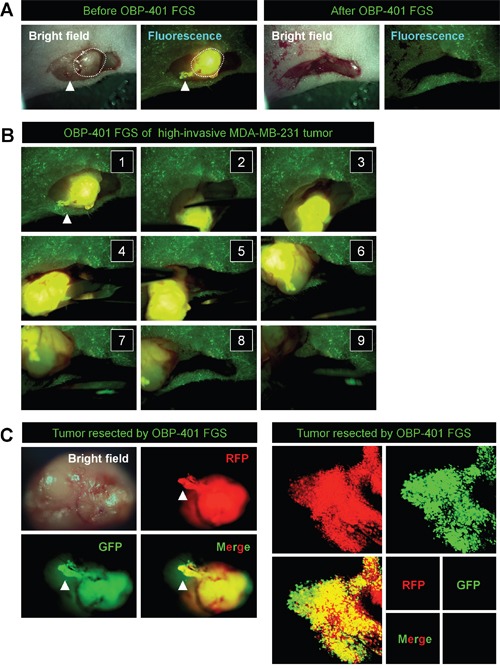
*In situ* OBP-401 GFP-labeling visualizes invading cancer cells and enables complete resection of high-invasive MDA-MB-231-RFP **A.** Representative high-magnification images of high-invasive MDA-MB-231-RFP before and after OBP-401 FGS using the hand-held fluorescence Dino-Lite scope. **B.** Step-by-step procedure of OBP-401 FGS for high-invasive MDA-MB-231-RFP (see Supplementary Movie S1). **C.** Representative images of *en bloc* tumor resected by OBP-401 FGS. Representative images of invading cancer cells resected by OBP-401 FGS visualized at the single-cell level using a confocal laser-scanning microscope (FV1000, Olympus, Tokyo, Japan).

In situ OBP-401 labeling enabled tumor margins to be visualized (Figures 2B, 2D) and enabled tumor resection without residual cancer cells (Figure 2E). The time course of the FGS procedure for the high-invasive MDA-MB-231 variant is shown in Figure 3, using the portable Dino-Lite fluorescence scope. Confocal imaging visualized the resected tumor at the single-cell level.

### OBP-401 enables detection and FGS of residual breast cancer after BLS

**Figure 4:**
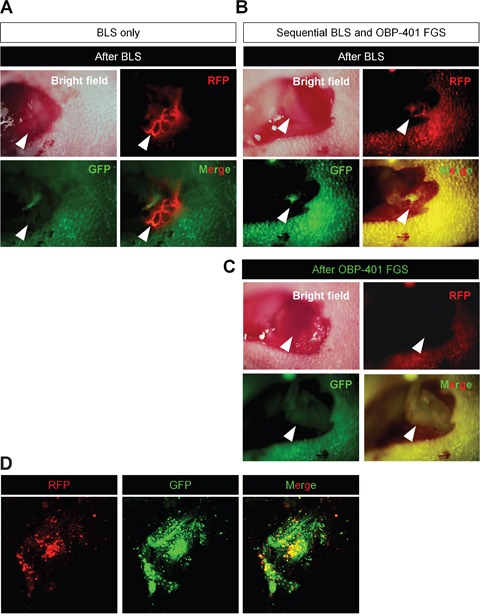
Sequential BLS and OBP-401 FGS of high-invasive MDA-MB-231-RFP OBP-401 was injected intaratumorally at 1 × 10^8^ PFU when tumors reached approximately 10 mm^3^ (diameter; 3 mm). The labeled orthotopic tumor was initially resected by BLS and then residual tumor was resected by FGS. **A.** Representative high-magnification images of invading cancer cells in a lymphatic duct after BLS. **B.** Representative macroscopic images of incomplete resection of the tumor after BLS (before OBP-401 FGS). **C.** Representative high-magnification images of surgical area after sequential BLS and OBP-401 FGS. **D.** Representative microscopic image of resected residual tumor after OBP-401 FGS acquired with confocal laser scanning microscope FV1000 (Olympus, Japan).

OBP-401 GFP labeling detected high-invasive MDA-MB-231-RFP cells invading a lymphatic duct not otherwise detected (Figures 4A, 4B). OBP-401 also enabled detection of residual breast-cancer cells at the single-cell level after BLS (Figure 4C). We were able to precisely remove the residual breast-cancer cells after BLS using OBP-401 FGS with a minimum resection area (Figure 4D).

### BLS results in recurrence and FGS does not

**Figure 5:**
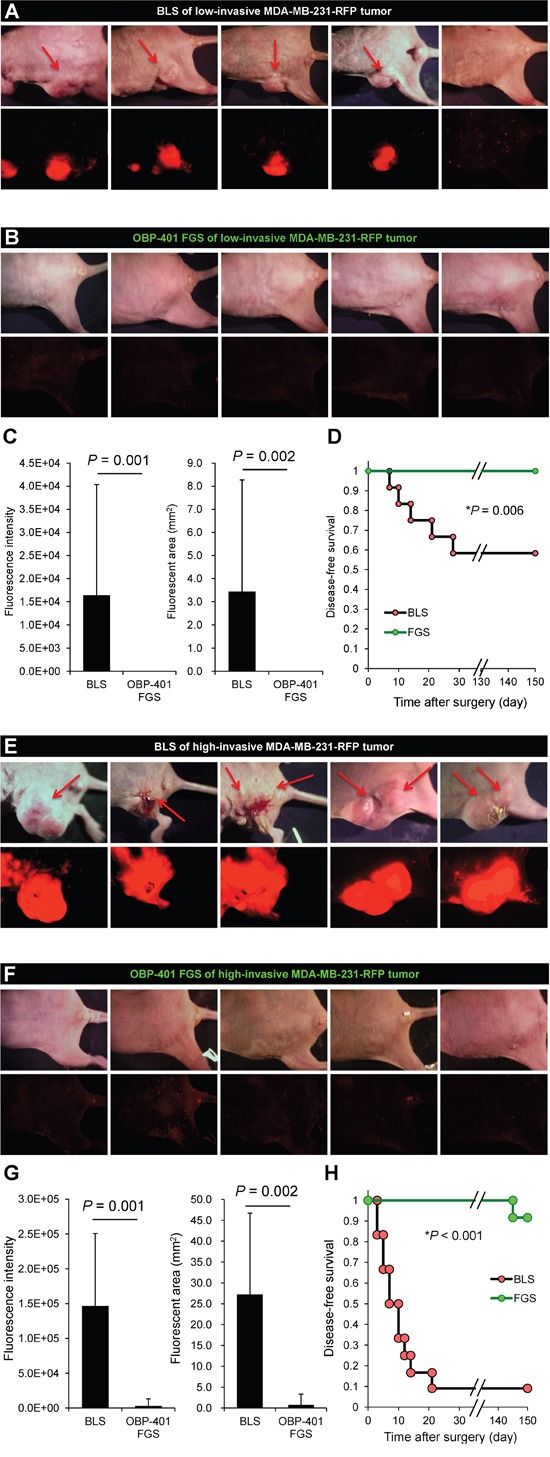
OBP-401-based FGS controls local recurrence of high- and low-invasive MDA-MB-231-RFP **A.** Representative whole-body images 150 days after BLS of low-invasive MDA-MB-231-RFP. Arrows indicate growing tumors. **B.** Representative whole body images 150 days after OBP-401 FGS for low-invasive MDA-MB-231-RFP. **C.** Comparison of fluorescent area of local recurrent tumors after BLS or OBP-401 FGS for low-invasive MDA-MB-231-RFP (right). Comparison of fluorescence intensity of local and metastatic tumors after BLS or OBP-401 FGS for low-invasive MDA-MB-231-RFP (left). **D.** Kaplan-Meyer shows disease-free survival after BLS or OBP-401 FGS for low-invasive MDA-MB-231-RFP. **E.** Representative whole-body images 120 days after BLS for high-invasive MDA-MB-231-RFP. Arrows indicate growing tumors. **F.** Representative whole-body images 120 days after OBP-401 FGS of high-invasive MDA-MB-231-RFP. **G.** Comparison of fluorescent area of local recurrent tumors after BLS or OBP-401 FGS for high-invasive MDA-MB-231-RFP (right). Comparison of fluorescence intensity of local recurrent tumors after BLS or OBP-401 FGS for high-invasive MDA-MB-231-RFP (left). **H.** Kaplan-Meyer curve shows disease-free survival after BLS or OBP-401 FGS for high-invasive MDA-MB-231-RFP. Fluorescent area and fluorescence intensity were calculated with ImageJ software. Data are shown as average ± SD.

We compared the rate of local recurrence after OBP-401 FGS or BLS alone for the high- and low-invasive MDA-MB-231-RFP tumor. With the low-invasive MDA-MB-231-RFP tumor after BLS, 5 of 12 mice recurred locally and 2 of 10 had lymph-node metastasis. With the high-invasive MDA-MB-231-RFP, 10 of 12 mice recurred locally and 10 of 12 also had lymph-node metastasis after BLS. After FGS, none of the 12 low-invasive MDA-MB-231-RFP recurred locally or distantly and, with the high-invasive MDA-MB-231-RFP, none recurred locally and 2 recurred distantly out of 14 total (Figure 5, Table 1).

**Table 1:** Efficacy of BLS or FGS on high- and low-invasive triple-negative breast cancer

Local recurrence	Positive	Negative	Lymph node metastasis recurrence	Positive	Negative
BLS Low-invasive	5	7	BLS Low-invasive	2	10
OBP-401 FGS Low-invasive	0	12*	OBP-401 FGS Low-invasive	0	12
BLS High-invasive	10	2	BLS High-invasive	10	2
OBP-401 FGS High-invasive	0	12**	OBP-401 FGS High-invasive	2	10***

* *P*=0.012;

** *P*<0.001;

*** *P*<0.001

### Comparison of survival

**Figure 6:**
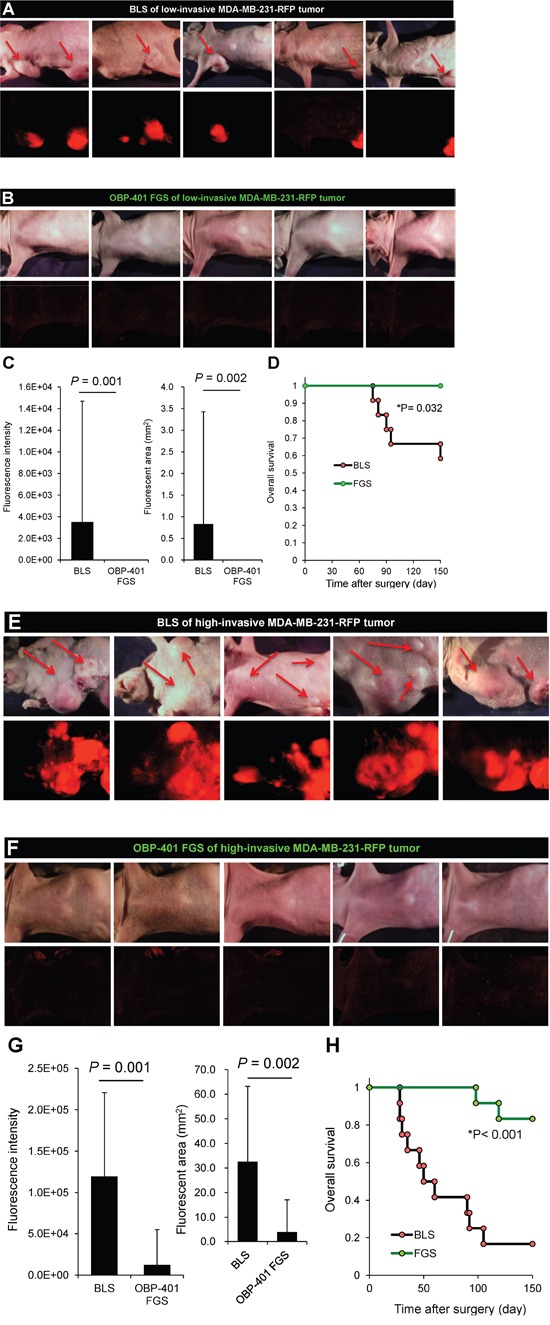
OBP-401-based FGS inhibits metastatic recurrence **A.** Representative whole-body images 150 days after BLS for low-invasive MDA-MB-231-RFP. Arrows indicate growing metastatie tumors. **B.** Representative whole-body images 150 days after OBP-401 FGS for low-invasive MDA-MB-231-RFP. **C.** Comparison of fluorescent area of lymph-node metastasis after BLS or OBP-401 FGS for low-invasive MDA-MB-231-RFP (right). Comparison of fluorescence intensity of lymph-node metastasis after BLS or OBP-401 FGS for low-invasive MDA-MB-231-RFP (left). **D.** Kaplan-Meyer curve shows the overall survival after BLS or OBP-401 FGS of low-invasive breast cancer. **E.** Representative whole-body images 120 days after BLS for high-invasive MDA-MB-231-RFP. Arrows indicate growing metastatic tumors. **F.** Representative whole-body images 120 days after OBP-401 FGS for high-invasive MDA-MB-231-RFP. **G.** Comparison of fluorescent area of lymph node metastasis after BLS or OBP-401 FGS for high-invasive MDA-MB-231-RFP (right). Comparison of fluorescence intensity of l lymph node metastasis after BLS or OBP-401 FGS for high-invasive MDA-MB-231-RFP (left). **H.** Kaplan-Meyer curve shows the overall survival after BLS or OBP-401 FGS of high-invasive MDA-MB-231-RFP. Fluorescent area and fluorescence intensity were calculated with ImageJ software. Data are shown as average ± SD.

After FGS with the low-invasive MDA-MB-231-RFP, all mice were alive at 150 days, in contrast to 60% of the mice receiving BLS. With the high-invasive MDA-MB-231-RFP tumor, 80% of the mice were alive at 150 days with FGS and only 10% with BLS (Figure 6).

In the present report, we demonstrate that high-invasive triple-negative breast cancer (TNBC) can be cured by OBP-401-based FGS, in contrast to standard BLS, which cannot cure this disease, in orthotopic nude mouse models. The present results demonstrate that recurrence of even a highly-invasive TNBC can be prevented in a clinically-relevant mouse model by FGS. Our results thereby indicate the clinical potential of OPB-401-based FGS for highly-aggressive cancer. OBP-401-based FGS can also be applied to liver metastasis resection [8] and other metastatic disease. FGS can also be performed with chemically-activable fluorescent probes [10–13] that can be used in a complementary manner with OBP-401 to ensure all cancer cells can be visualized by the surgeon, including recurrent disease.

Previously developed concepts and strategies of highly-selective tumor targeting can take advantage of molecular targeting of tumors, including tissue-selective therapy which focuses on unique differences between normal and tumor tissues [14–19].

## MATERIALS AND METHODS

### Animal experiments

Athymic nude mice (AntiCancer Inc., San Diego, CA) were used in this study. Mice were fed with autoclaved laboratory rodent diet (Tecklad LM-485, Western Research Products). All animal studies were conducted in accordance with the principals and procedures outlined in the National Institutes of Health Guide for the Care and Use of Laboratory Animals under assurance A3873-01. All animal studies were conducted with an AntiCancer Institutional Animal Care and Use Committee (IACUC)-protocol specifically approved for this study. In order to minimize any suffering of the animals, anesthesia and analgesics were used for all surgical experiments. Animals were anesthetized by intramuscular injection of a 0.02 ml solution of 20 mg/kg ketamine, 15.2 mg/kg xylazine, and 0.48 mg/kg acepromazine maleate. The response of animals during surgery was monitored to ensure adequate depth of anesthesia. Ibuprofen (7.5 mg/kg orally in drinking water every 24 hours for 7 days post-surgery) was used in order to provide analgesia post-operatively in the surgically-treated animals. The animals were observed on a daily basis and humanely sacrificed by CO_2_ inhalation if they met the following humane endpoint criteria: prostration, skin lesions, significant body weight loss, difficulty breathing, epistaxis, rotational motion and body temperature drop. The use of animals was necessary to develop fluorescence-guided surgery of highly-metastatic triple-negative breast cancer. Mice were housed with no more than 5 per cage. Mice were housed in a barrier facility on a high-efficiency particulate arrestance (HEPA)-filtered rack under standard conditions of 12-hour light/dark cycles. The animals were fed an autoclaved laboratory rodent diet.

### GFP-expressing telomerase-specific adenovirus

The recombinant GFP-expressing, cancer-specific, replication-selective adenovirus vector OBP-401, has the promoter element of the human telomerase reverse- transcriptase (*hTERT*) gene which drives the expression of E1A and E1B genes linked to an internal ribosome entry site for selective replication only in cancer cells. The *GFP* gene is driven by the CMV promoter. The virus was constructed and produced as previously described [2–9, 20, 21].

### Cell culture

The human triple-negative breast cancer cell line, MDA-MB-231 [22, 23] was maintained and cultured in DMEM with 10% fetal bovine serum (FBS) and 5% penicillin/streptomycin. Highly-metastatic MDA-MB-231 cells, expressing red fluorescent protein (MDA-MB-231-RFP), derived from a lymph-node metastasis after orthotopic transplantation in nude mice were also maintained and cultured in DMEM with 10% fetal bovine serum (FBS) and 5% penicillin/streptomycin.

### *In vivo* whole body/whole-tumor and cellular imaging

The OV100 small animal imaging system (Olympus Corp., Tokyo, Japan), was used. The OV100 contains an MT-20 light source (Olympus Biosystems, Planegg, Germany) and DP70 CCD camera (Olympus), for subcellular imaging in live mice. The optics of the OV100 have been specially developed for macroimaging as well as microimaging with high light-gathering capacity. The instrument incorporates a unique combination of high numerical aperture and long working distance. Four individually-optimized objective lenses, parcentered and parfocal, provide a 105-fold magnification range for seamless imaging of the entire body down to the subcellular level without disturbing the animal [24]. The FV1000 scanning laser confocal microscope (Olympus, Tokyo, Japan) was used for cellular imaging of resected tumors [25].

### OBP-401 based fluorescence-guided surgery (OBP-401 FGS)

All animal procedures were done under anesthesia using s.c. administration of a ketamine mixture (see above). After skin incision, the OBP-401-GFP-labeled tumor was visualized with the OV100 [2–9, 20, 21]. The tumor was resected under fluorescence-guidance using the Dino-Lite (AM4113T-GFBW Dino-Lite Premier; AnMo Electronics Corp., Hsinchu, Taiwan), a hand-build scope [26]. After tumor resection, if there was residual tumor, a second resection was performed. After surgery, the skin incision was closed with a 6-0 suture.

### Statistical analysis

Data are shown as means ± standard deviation (SD). For comparison between two groups, significant differences were determined using Student's t-test. *P* values of < 0.05 were considered significant. For comparison of more than two groups, statistical significance was determined with a one-way analysis of variance (ANOVA) followed by a Bonferroni multiple group comparison test. Pearson chi-square analysis was used to evaluate the rate of local recurrence, lymph node metastasis between BLS and OBP-401 FGS of parental MDA-MB-231 and high-metastatic MDA-MB-231 tumors. Statistical analysis for disease-free survival and overall survival was performed using the Kaplan-Meier test along with log-rank test.

## SUPPLEMENTARY VIDEOS




