# Supplementary material for: Fluorescence-guided surgery of a highly-metastatic variant of human triple-negative breast cancer targeted with a cancer-specific GFP adenovirus prevents recurrence
Source: Oncotarget. 2016 Sep 28;7(46):75635–47. doi: 10.18632/oncotarget.12314 (PMC5342766; doi:10.18632/oncotarget.12314)
Supplement: Supplementary file 1 [file oncotarget-07-75635-s001.pdf]

Fluorescence-guided surgery of a highly-metastatic variant of human triple-negative breast cancer targeted with a cancer-specific GFP adenovirus prevents recurrence

SUPPLEMENTARY VIDEOS

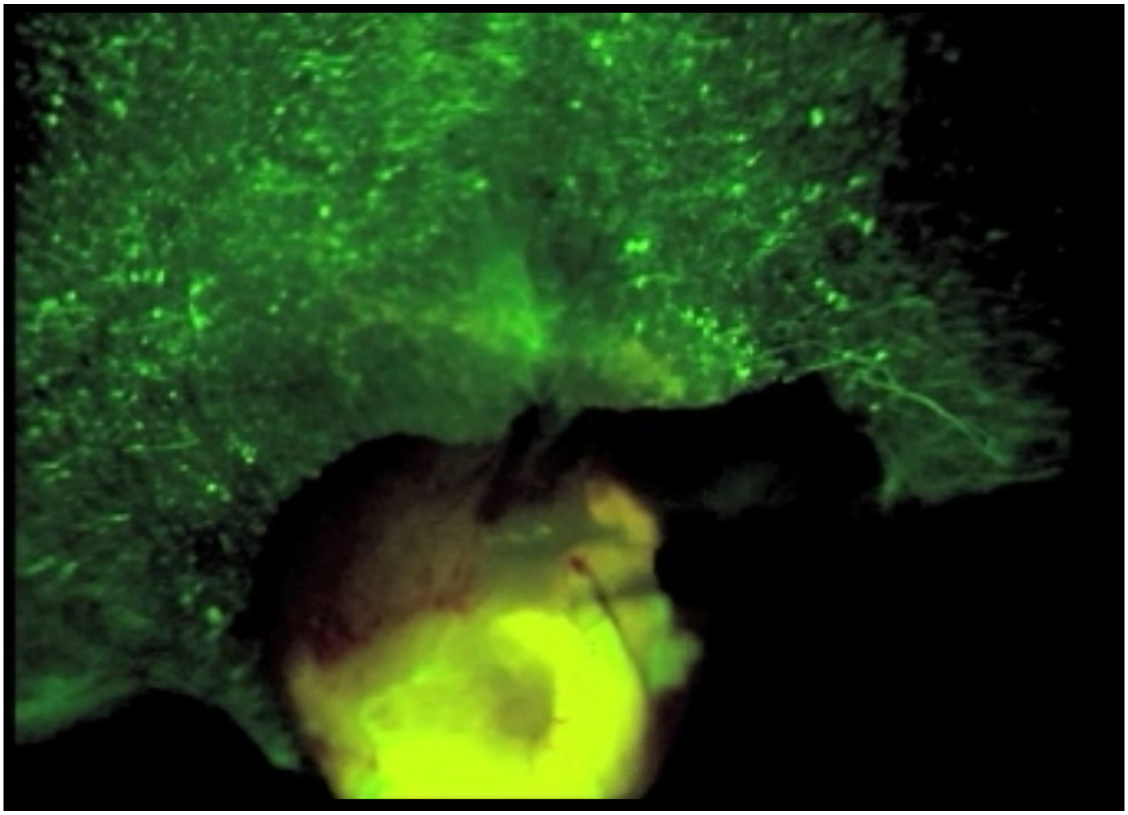

Supplementary Movie S1: OBP-401 based fluorescence-guided surgery for high-invasive MDA-MB-231-RFP. Orthotopic MDA-MB-231-RFP breast tumor was resected using the hand-held fluorescence Dino-Lite scope.
